# The Epidemiology of Suicide in Young Men in Greenland: A Systematic Review

**DOI:** 10.3390/ijerph15112442

**Published:** 2018-11-01

**Authors:** Hannah Sargeant, Rebecca Forsyth, Alexandra Pitman

**Affiliations:** 1 UCL Division of Psychiatry, London W1W 7NF, UK; hannah.sargeant.11@ucl.ac.uk (H.S.); rebecca.forsyth.14@ucl.ac.uk (R.F.); 2 Camden & Islington NHS Foundation Trust, London NW1 0PE, UK

**Keywords:** suicide, premature mortality, young men, Greenland, Denmark

## Abstract

Suicide is the leading cause of death among young men aged 15–29 in Greenland, but few epidemiological studies have described this problem. We aimed to summarise descriptive epidemiological studies of suicide in young men in Greenland compared with other demographic groups in Denmark and Greenland to inform future suicide prevention strategy. We searched PubMed, PsycINFO, and Embase using an agreed search strategy to identify English-language papers describing suicide epidemiology in Greenlandic men aged 15–29. We followed PRISMA guidelines in screening and appraising eligible publications. Eight articles fulfilled inclusion criteria of 64 meeting search criteria. Findings covering 1970–2011 supported a dramatic rise in suicide rates in Greenlandic men aged 15–24 from 1976, who remained the highest-ranking demographic group over 1976–2011 compared with men and women of all age groups in Denmark and Greenland. Highest rates recorded were almost 600 per 100,000 per year in men aged approximately 20–23 over 1977–1986. No studies described suicide epidemiology after 2011, and no studies described risk factors for suicide in young men. Given the very high suicide rates recorded for young men over 1976–2011, such studies will be essential for informing the development and evaluation of appropriate preventive interventions.

## 1. Introduction

Internationally suicide is the second leading cause of mortality in young men after accidental deaths [1]. Mortality indicators such as potential years of life lost (PYLL) demonstrate the economic and social cost of suicide in young men, which has become a serious public health problem over the last 70 years [1]. Despite widespread international recognition of the problem [1], and growing media concern, research is lacking on the mediators of suicide risk in young men and the interventions to mitigate them. International studies suggest that individual-level risk factors for suicide in men include psychiatric illness, substance misuse, lower socioeconomic status, rural residence, and single marital status [1]. However, there is a clear need for epidemiological studies of young men in specific regions, to understand the local sociocultural influences on their suicide risk, and the development of appropriate responses. The suicide rate in Arctic communities is considered a major public health concern [2], particularly in adolescents and young men [3]. Indigenous communities in the circumpolar north face a range of geocultural and economic hardships, including the challenges of inadequate housing and access to health care, in the context of global forces eroding local traditions [2]. Greenland has attracted particular media concern over suicide rates in young men, but few epidemiological studies have described this problem. A large (2,150,000 km^2^) and remote island near the continent of North America, Greenland is a former colony of Denmark, largely populated by Inuit people [4]. According to suicide data published by Statistics Greenland, suicide accounts for 8% of total deaths in Greenland and is the leading cause of death among young men aged 15–29 [5]. However, due to its territorial status, Greenland’s suicide rates are subsumed within those for Denmark, such that international rankings mask the problem. Broad comparison of the population suicide rates published by Statistics Greenland for 2011 (83 per 100,000) [5] greatly exceed those published by the World Health Organisation (WHO) for Guyana, the country with the highest population suicide rates internationally that year (32.5 per 100,000) [6], or for the 2015 global average of 10.7 per 100,000 [7]. International comparisons of suicide rates in young men identify particularly high rates in Eastern Europe and Japan, suggesting that suicide risk is higher for young men in countries undergoing transition or rapid social change [8]. Societies in transition experience changes in cultural norms, family cohesion, economic pressures, substance use, and migration patterns. The complex interaction of these variables requires investigation at the local level.

Greenland proposed a Greenlandic suicide prevention strategy in 2004, identifying young men as a high risk group, but acknowledging a lack of research into the aetiology of suicide in this group [9]. Although suicide prevention was included in the 2007–2012 Greenlandic public health programme, it was omitted in the 2013–2019 version [10]. However, Greenland participates in a US-led 2015 Arctic Council project, RISING SUN (Reducing the Incidence of Suicide in Indigenous Groups: Strengths United through Networks) to share expertise in suicide prevention in Arctic communities [2]. The success of such policy efforts relies in part on having a clear understanding of the problem epidemiologically. Suicide data collection in Greenland has a broken history, and policy-makers lack a clear picture of recent trends and high-risk groups. The earliest systematic suicide data collection in Greenland was by one physician from 1891–1930 [11], but there is then a gap until 1951 when annual reports by the country’s Chief Medical Officer commenced [12]. Whilst actual numbers of suicides are published by Statistics Greenland for 1990–2013 [5], comparison of patterns with other Arctic areas is difficult without clear presentation of rates, both temporally and by age group. To inform future Greenlandic suicide prevention strategies and highlight gaps in evidence, it is important to understand how suicide rates in young men in Greenland compare with those for young men in Denmark, and with men in other age groups in Greenland.

We aimed to conduct a systematic review of research studies describing the epidemiology of suicide in young men (aged 15–29) in Greenland compared with young men in Denmark, and Greenlandic men in other age groups, using international evidence published up until 2018. In synthesising these findings we aimed to test the hypothesis that suicide rates in young men in Greenland are greater than those for these other groups. Our objectives were to describe historic and recent temporal trends in suicide rates in young men, comparing rates with those for other age and gender groups within Greenland and with other young men in Denmark, and identifying specific risk factors for suicide in young men. In conducting our review we identified a dramatic rise in suicide rates in Greenlandic men aged 15–24 from 1976, such that young men supplanted older men as the highest-risk demographic group for suicide over the period 1976–2011. Our search criteria did not identify any studies describing suicide epidemiology in young men from 2011 onwards, nor specific risk factors for suicide at any point, so recent patterns remain unclear.

## 2. Materials and Methods

### 2.1. Search Strategy

In developing our search criteria we decided to exclude research on self-harm due to wide international variations in definitions based on degree of suicidal intent [13], although acknowledging that self-harm is a key risk factor for suicide in men in high-income countries [14]. We chose the age range 15–29 to cover WHO definitions of youth (aged 15–24 years), teenagers (aged 15–19 years), and young adults (aged 20–24 years) [1], as well as the upper limit of under 30 used in the Greenlandic suicide prevention strategy [9]. We registered our review protocol on PROSPERO register of systematic reviews, and used the PRISMA checklist to guide the design and reporting of our study [15].

We searched the online database, PubMed, using the MeSH terms “Suicide” and “Greenland” and the equivalent keywords Suicid* and Greenland, to give the final search criteria ((“Suicide” [Mesh]) OR Suicid*) AND (“Greenland” [Mesh] OR Greenland). We used no date restrictions on publication period. The search was repeated on Embase, and with slight variation on PyscINFO as the MeSH term “Greenland” was not available. No restrictions were applied to age-range or gender, as the predicted relatively limited number of articles on this topic made it feasible to apply age and gender exclusions post-search. We conducted the search on 11 October 2017 and screened titles and abstracts for eligibility, followed by full text review. We conducted secondary searching of the reference lists of identified articles, and of the publications lists of identified authors, and emailed international experts in the field to identify additional references. We repeated the search in PubMed on 15 August 2018 to check for any recent studies. 

### 2.2. Inclusion and Exclusion Criteria

We included articles:

- reporting primary quantitative research involving any length of follow-up
- with titles/abstracts mentioning suicide, fatal or non-fatal suicide attempts, or suicidal ideation
- with titles or abstracts mentioning the Greenlandic population or Inuit population
- specifying age-ranges covering men aged 15–29
- specific to the Greenlandic population (those born in Greenland with at least one Greenland-born parent)
- published in English

We excluded articles:

- specific to Inuit populations in other countries
- presenting data on the age-range 15–29 but not specific to men
- focusing on non-fatal suicide attempt or suicidal ideation but not suicide

### 2.3. Study Selection

Two authors (AP and HS) independently screened all citations identified in the search for eligibility, comparing included/excluded articles for agreement. For those identified for full text review, two authors (AP and HS/RF) reviewed each manuscript independently to rate eligibility. The third independent reviewer resolved any discrepancies arising during title, abstract or full-text review.

### 2.4. Data Extraction

We developed a data extraction form based on STROBE criteria for observational studies [16]. For each article the following data were extracted: design, objectives, setting, participants, outcomes, summary measures relevant to research question (e.g., absolute numbers of suicides, suicide rates, standardised mortality ratios, other risk estimates), bias and limitations (as a marker for quality), and interpretation. For each paper included in the study, two authors (AP and HS/RF) independently reviewed each paper, rating quality (including an assessment of study and outcome bias) and summarising findings, including all summary measures presented. Each reviewer completed a data extraction form independently, and compared findings, updating entries in a master data extraction form. Again, a third independent reviewer resolved any discrepancies. As a team we discussed the potential for publication bias, and the potential for selective reporting within studies.

Due to the mixture of summary measures presented, the differing age-groupings, and the discontinuous time periods covered, we used a narrative approach to synthesise temporal findings, taking into account study quality. We used the PRISMA statement to structure findings [17].

## 3. Results

### 3.1. Studies Identified

**Figure 1:**
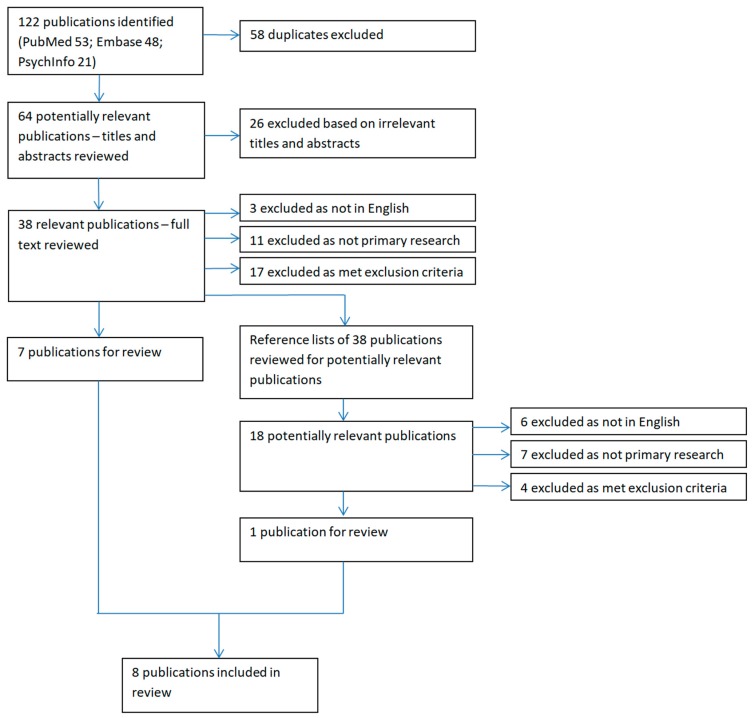
Flow diagram summarising the study selection process.

Eight articles fulfilled inclusion criteria, of 64 meeting search criteria (Figure 1). Included studies were published between 1979 and 2015, with characteristics summarised in Table 1. Studies reported suicide data specific to young men over 1970–2011. There was great heterogeneity of time periods studied, ranging from rates for single years, to an aggregated period of 41 years (1970–2011) [18]. There was also great heterogeneity of measures presented (absolute numbers; suicide rates per 100,000 inhabitants; suicide rates per 100,000 person-years), prohibiting meta-analysis. Whilst one study used a case-control design [19], the remaining seven presented cross-sectional data derived from national suicide registers (based on death certificates and police reports), or from these primary sources themselves. Six of those seven aggregated data for the periods of interest, but one presented data for seven specific years over 1970–1995 [20], although but only for West Greenland where suicide rates are lower than for East Greenland or the capital Nuuk [21]. Seven studies reported suicide data on Greenlandic individuals of all ages, from which we were able to extract selected findings relating to men aged 15–29. One study concentrated solely on young people aged 15–30 [22]. Two only reported data only from West Greenland [20] or Nuuk [19].

### 3.2. Study Quality

The quality of included studies was partly a function of the quality of routine data available, which improved over the years studied. The measures presented also became more meaningful over time; from absolute numbers to rates per 100,000 inhabitants/person-years. Both of these improvements are demonstrated in the chronological presentation of papers in Table 1. For example an early study presented absolute numbers of suicides by age group and gender for 1972 and 1973, but no suicide rates specific to men (or indeed men in specific age groups) [19]. In later studies crude suicide rates were represented as per 100,000 inhabitants [12,22,23] or per 100,000 person-years [18,20,21]. None of the studies used join-point regression models to calculate annual percentage change statistics. Many studies compared suicide rates in different age or gender groups (or between cases and controls) without providing test statistics. Studies investigating risk factors for suicide aggregated genders or age groups, probably for reasons of power, or lacked test statistics.

### 3.3. Risk of Bias across Studies

The dramatic rise in suicide rates in the early 1970s, increasing from zero in young men aged 15–24 in West Greenland in 1970 to 249 per 100,000 in 1976 [20], may have reflected early under-reporting or misclassification bias. In 1970 all reported suicide cases in West Greenland were for men in the age group 35–59 years of age, but by 1976 suicides were recorded across all age groups above 15 [20]. At the population level, official Greenlandic figures for 1967–1971 indicated an average of 6.4 suicides per year (16.7 per 100,000 Greenlandic-born inhabitants), which had risen to 21.6 per year (53.9 per 100,000) for 1972–1976 [23]. Due to the high relative number of accidents and unidentified causes of death in Greenland, it is possible that a substantial number of suicides are misclassified as accidents [24]. Cultural reasons may underlie the differential recording by age group of suicide *versus* accidental death on the cause of death register, and temporal variations in such practices. The only evidence of selective reporting within studies was for the specific risk factors investigated, which may have reflected inductive bias.

### 3.4. Results of Individual Studies

#### 3.4.1. Aggregated Data for Specific Periods

Over the aggregated period 1970–2011, the highest suicide rates were in men aged 20–24 (at around 410 per 100,000 person-years), with a clear excess of male suicides [18]. Suicide rates for men for the 41 year period 1970–2011 fell with advancing age group, to their lowest level in men aged 65–69 (at around 50 per 100,000 person-years), then increased slightly for men over 70 [18]. These figures are consistent with aggregated suicide data for a shorter 11 year period (1974–1984), showing that suicide rates in young men aged 20–24 were the highest of all age and gender groups in Greenland (at 387 per 100,000 inhabitants per year) compared with 151 per 100,000 for men aged 15–19, 162 per 100,000 for men aged 25–39, and 73 per 100,000 in men over 40 [12]. The gender gap in suicide rates over this period (1974–1984) was greatest in the age group 20–24 (387 for men *versus* 94 for women per 100,000 inhabitants) [12]. 

Similarly, aggregated suicide data for the shorter 10 year period 1977–1986 show a consistent pattern of highest suicide rates in Greenlandic men aged approximately 20–23, greatly exceeding those for women of the same age [23]. Visual plots presented for average suicide rates per 100,000 population per year showed that suicide risk increased sharply from the ages of approximately 15–17, peaking at almost 600 per 100,000 per year at approximately age 20–23, falling thereafter but with further peaks aged around 35 (approximately 300 per 100,000) and around 55 (approximately 250 per 100,000). A great disparity between male and female suicide rates was apparent from the age of approximately 17–27 [23]. 

By the 1990s, aggregated data for the narrower time periods 1990–1999 [21] and 1990–1995 [24] reveal men aged 15–24 to have been the highest risk group. A study presenting visual plots of age-specific suicide rates for the aggregated period 1990–1999 showed suicide rates to be highest in men aged 20–24 in Greenland (approximately 470 per 100,000 person years), closely followed by men aged 15–20 (approximately 430 per 100,000 person years); both considerably higher than those for men or women in other age groups in Greenland, or for men and women of any age in Denmark. This disparity continued up until approximately the age of 45, when rates in men and women in Greenland and Denmark started to converge at around 50 per 100,000 person-years [21].The pattern of suicide risk in Danish men and women over the same period was very different; rising slowly across the age groups but remaining below approximately 70 per 100,000 person-years [21]. In Denmark there was also a much lower ratio of male:female suicides of 1.8 compared to 4.3 for Greenland [21].

Aggregated data for the shortest span of 1990–1995 show that for this period men aged 15–19 had overtaken men aged 20–24 as the group at highest risk, these being the two highest ranking age groups for suicide rates in all age-groups in either gender [24]. Rates for men aged 15–19 during 1990–1995 were approximately 480 per 100,000, declining with age at approximately 440 per 100,999 for men aged 20–24, and 300 per 100,000 for men aged 25–29, and declining thereon. This compared with age-standardised population rates of nearly 110 per 100,000 population over that period. Higher rates for men than women applied in all age groups [24].

#### 3.4.2. Temporal Trends

Only one study described temporal changes in suicide rates for young men, reporting rates for seven specific years over 1970–1995, but solely for West Greenland and using relatively wide age bands [20]. This recorded a zero suicide rate for young men aged 15–24 in 1970, and all reported suicide cases in men that year were within the age group 35–59 years [20]. By 1976 suicide rates in men aged 15–24 had risen dramatically to 249 per 100,000, becoming the group at highest risk, ranking above those for men aged 25–34 at 123 per 100,000 [20]. They remained the highest risk group at all remaining data points (1982, 1987, 1990, 1993, 1995), having peaked at 577 per 100,000 in 1990, and fallen to 238 per 100,000 in 1995 [20]. Throughout this period, men aged 25–34 also had high suicide rates, reaching a maximum of 297 per 100,000 in 1990 (compared with 577 per 100,000 for men aged 15–24). However, by 1995 the disparity between high suicide rates in men aged 15–24 and those aged 25–34 was less marked, at 238 *versus* 219 per 100,000 [20].

#### 3.4.3. Period Effects

Gender-specific suicide data on cohorts born from 1948–1978 provide evidence that suicide rates in the age group 15–29 rose in successive cohorts born from 1952, matching the onset of major sociocultural change. For example suicide rates in men aged 15–29 born in 1952 were approximately 4 per 100,000 births in that cohort, rising to approximately 60 per 100,000 births in the 1978 birth cohort [21]. Whilst this is suggestive of a period effect, we lack comparative data describing other age groups in those cohorts [25].

#### 3.4.4. Regional Variation

One study presented regional variations in youth suicide from 1970–1999, which were not broken down by gender [21]. However, this suggested that young people in remote areas of East Greenland had generally higher suicide rates, rising from approximately 200 per 100,000 person-years in 1970–1974, to approximately 800 per 100,000 person-years in 1995–1999. Meanwhile, youth suicide rates peaked in the capital, Nuuk, in 1980–1984 at above 300 per 100,000 person-years, and fell to between 100 and 200 per 100,000 person-years in 1995–1999. Suicide rates for young people in West Greenland were lowest among regions but rose throughout the 1970s and 1980s, overtaking those in Nuuk in 1985–1989 where they plateaued at approximately 200 per 100,000 person-years throughout the 1980s and 1990s. As the majority of these youth suicides will have been in men, only tentative conclusions can be drawn about these patterns of high suicide rates in rural areas applying to males aged 15–29 [21].

#### 3.4.5. Risk Factors

We were unable to identify specific risk factors for suicide in young men in Greenland because analyses of suicide risk factors aggregated all age groups or both genders, or lacked formal statistical tests. One study investigating seasonality found a significant midsummer peak for men of all ages, but this was not specific to young people [20]. One study, described briefly here, provided limited findings on occupational groups and triggering factors [22]. This reported absolute numbers and proportions of suicides in young men by occupation and triggering factors, for the aggregated period 1982–1986 [22], but did not use rates or statistical tests (perhaps due to relatively low numbers). Only tentative inferences can be made from their finding that 78% of suicides among youth aged 15–30 were in men, and that these men primarily worked in traditional hunting/fishing jobs, unskilled jobs, or were unemployed [22]. Using witness statements from police reports, this study also found that rejection by friends or parents “and other shameful situations” were implicated in 20% of male suicide cases in the age group 15–30 but comparisons with other groups were not presented [22]. One other study found that for all male suicides, 16% of death certificates mentioned alcohol dependence/intoxication, but lacked data on age groups [20], so again little can be inferred about young men.

#### 3.4.6. Suicide Methods

As data on suicide methods in the identified studies did not disaggregate data by age-group and gender we could not identify the methods used by young men in Greenland, or temporal trends in these methods. From 1968–1995, 93% of suicides in the whole population were violent, predominantly shooting or hanging, and this was more common in men than women (96% *versus* 81%; no test statistic provided) [20].

## 4. Discussion

### 4.1. Main Findings

Overall our findings support a pattern of consistently higher suicide rates in young Greenlandic men over the period 1976–2011, both when compared with men in other age groups, same-age Greenlandic women, and with men and women of all age groups in Denmark [21]. Aggregated data for periods over 1970–2011 suggests that men aged 20–24 were the highest risk group for suicide in Greenland, followed by men aged 15–19. Evidence from the narrowest period of reporting in 1990–1995 suggests that men aged 15–19 replaced men aged 20–24 as the group at highest risk around this point, at approximately 480 per 100,000 population [24]. This was also the point at which population rates stabilised, at approximately 110 per 100,000 population until the end of 1995 [24]. However, as the only study reporting rates for single years collapsed both age ranges [20] we cannot pinpoint if and when men aged 15–19 overtook men aged 20–24 as the highest ranking age group for suicide.

At the Greenlandic population level, age-standardised population suicide rates over the period 1972–1995 increased markedly from approximately 44 per 100,000 population in 1975 to approximately 110 per 100,000 in 1984–1989, stabilising at 110 per 100,000 from 1990–1995 [24]. This dramatic rise coincides with a time of high suicide rates in Greenlandic men aged 15–24, [12,18,20,21,23,24] suggesting that this age group accounted for the population-level transition. This apparent shift in suicide rates in Greenland from elderly to younger men matched the transition that occurred from 1950–1999 in some high-income countries [1], as suicide rates rose in middle-aged men (aged 35–45) and in young men (aged 15–25), largely supplanting older men as the group at highest risk of suicide [26]. The studies we identified, however, did not present a fine-grained picture of whether and how this age transition occurred. Variation in the years and age ranges reported also hamper direct comparisons of suicide rates across age groups in Greenland to those in other countries.

Geocultural factors are likely to be relevant in explaining suicide epidemiology in Greenland. Primarily covered by an ice sheet, it is sparsely populated by approximately 57,000 people, mainly Inuit people who started to migrate there from Canada in 2500 BC [4]. Its high latitude, ranging from 59° N to 84° N, accounts for extreme seasonal variations in daylight and darkness hours. The country has arctic climatic conditions; seasonal temperatures vary with latitude and distance from the coast, but average yearly temperatures remain below 10 °C [27]. Its remoteness kept it culturally insulated from its neighbours until the 18th century, but in 1953 its colonial status ended and it became an integrated constituency, governed by the Danish state and giving Greenlanders Danish citizenship [28]. This marked the start of a period of rapid sociocultural and socioeconomic changes under the modernising influence of Denmark. These included a change from subsistence hunting and fishing to a wage-earning economy, the migration of non-Inuit people to Greenland, and increased urbanisation, influencing changes in infrastructure and housing [29]. Genetic studies find that over 80% of Greenlanders have some European ancestry due to recent migration (approximating to 25% of their genome), inherited primarily from male Europeans [4]. In 1979, Greenlanders voted for Home Rule, which gave them the right to elect their own parliament, reducing the influence of Denmark. Greenland gained further autonomy in 2008, when a vote for self-governance was passed by a 75% majority and Home Rule was replaced. Greenlanders are now recognised as separate people under international law, and the native Inuit language of west Greenland has official status in place of Danish. The country has autonomous control over areas such as education, health and environment, whilst Denmark still governs justice affairs, national security, civil rights and financial sectors [28].

These geopolitical factors set important context. The dramatic increase in suicide rates in young men in Greenland in the 1970s coincided with a period of rapid modernisation and social change. However, we were unable to identify specific risk factors for suicide in young men as no studies reported these. We can only therefore hypothesise that specific aspects of modernisation increase risk. Superficially, evidence for regional variation in male suicide rates appears to undermine arguments for the effects of modernisation, given that rates are much higher for men of all ages in rural areas of East and North Greenland than in West Greenland, or the capital, Nuuk [18] and for young men in rural East Greenland from 1968–1999 [21]. However, the explanation may lie in disparities in socioeconomic status between men in urban and remote areas. The problem is that the individual-level suicide risk factors identified for young men in other countries (psychiatric disorder, substance misuse, occupational group, ethnicity, rural residence, lower socioeconomic status, single marital status) have not been specifically tested in young men in Greenland, nor have the population-level risk factors (unemployment, social deprivation, media influences) identified in high-income countries [1]. Other explanations for marked rises among young men at the start of this period include improved reporting during the 1970s, misclassification bias, and the effects of rapid social change.

### 4.2. Findings in the Context of Other Studies

Whilst studies we identified covered the period 1970–2011, insights into suicide patterns in Greenland during earlier periods are provided by studies meeting our exclusion criteria. The Danish physician, Dr. Alfred Bertelsen, recorded 14 suicide cases from 1891–1930 [11]. His findings, published in Danish but reported elsewhere [12], were that eight cases were men, six were women, half the men were under 35 years of age, while all the women were over 35. In cases where he was able to conduct psychological autopsies he ascertained that all had been diagnosed with mental disorders [11]. His estimates of average annual suicide rates were 4 per 100,000 for the period 1891–1903 and 3 per 100,000 for the period 1901–1930, noting that the high comparative rate for accidental deaths suggested misclassification of suicides [12]. A 1955 anthropological study concluded that, at that time, suicide deaths largely occurred in elderly people who were no longer economically active, did not wish to burden their families, and attempted suicide only after consultation with family members [30]. Our findings for the period following this, coinciding with a period of rapid sociocultural and socioeconomic change, suggest a marked shift in risk towards much younger age groups.

Studies specifically describing suicide epidemiology in young men are lacking worldwide, and wide variation in the format of mortality statistics creates problems in comparing temporal patterns by age and gender group [1]. However, there is evidence that indigenous group status predicts high suicide rates in young men in regions neighbouring Greenland; specifically indigenous Sami in Arctic Norway [31], Native American men aged 15–24 in the US [32], and Inuit men aged 15–24 in Canada [33]. A study of men aged 15–34 years in small Alaskan communities from 1980–2007 found that community-level characteristics such as remoteness, fewer non-Natives, and cultural divides had higher suicide risks, whilst those with higher incomes, more married couples, and traditional elders had lower risks [34]. This is consistent with evidence that rural or remote residence is associated with risk of suicide in young men in Denmark, with explanations relating to the migration of healthy workers to cities, and the increasing socioeconomic disparity between men in rural and urban areas [35]. Analysis of data from Danish longitudinal psychiatric registers shows that Greenlandic men aged 15–24 have significantly higher first psychiatric admission rates than Danish men of the same age [36]. Psychiatric disorder is therefore implicated as one explanation for high suicide rates in young men in Greenland compared with their Danish counterparts.

### 4.3. Strengths and Limitations

#### 4.3.1. Strengths

We used a clear research question, and a comprehensive search strategy, with methods to identify unindexed papers. We registered our study protocol with PROSPERO, followed STROBE and PRISMA guidelines when conducting our review, and independently screened and critiqued papers. 

#### 4.3.2. Limitations at Study Level

These have largely been covered under study quality. A major limitation for many studies was that of statistical power, which may explain why included studies provided very limited data on risk factors by gender and age-group, and why many studies aggregated successive years of data. Presenting average age and gender-specific suicide rates for periods of up to 41 years obscured any year-on-year changes and made it hard to identify short-term variations during a period of rapid social change [18]. Even where gender- and age-specific suicide rates were presented for specific years over the period 1970–1995, these lacked statistical tests for time trends, and only related to West Greenland [20] so were unlikely to be generalisable to the rest of Greenland [21]. Without join-point regression models to calculate annual percentage change statistics we lacked an understanding of the magnitude and direction of short-term and long-term trends in age-specific suicide rates.

Misclassification bias was another general potential problem. All studies derived suicide cases from population death registers, or from the death certificates and police reports on which those registers were based. Only one was questionable in terms of whether it used comprehensive methods of identifying all cases [19]. In one included study, 94% of deaths from 1968–1999 (as recorded in death certificates) had been certified by a physician and issued with an underlying International Classification of Diseases (ICD) code for cause of death [21]. This highlighted that where no diagnosis is made or where classified as other injuries, it is possible that there was under-recording of suicide, which may have been differential by age group for cultural reasons. Given high rates of accidental death in young men, and wide international variation in the quality of suicide data, the great potential for underestimating, through misclassification, suicide deaths in young men has been acknowledged [1]. It is possible that high rates of suicide in young men in included studies might be under-estimates. Conversely, it is possible that biases might lead to the deaths of young men being more likely to be classified as suicide than deaths in other demographic groups. Validation studies, however, suggest that registration within official Greenlandic statistics is generally reliable [37].

#### 4.3.3. Limitations at Review Level

Due to our eligibility criteria we may have overlooked studies published in Danish or Greenlandic, and our search for unpublished studies may have been incomplete, thereby reflecting publication bias. By confining our study to studies reporting suicide rates we lacked the context of age and gender patterns in suicidal ideation and suicide attempts over a similar period, particularly where they provided gender [38] or cross-national [39] comparisons. 

### 4.4. Policy Implications

Although the studies we identified suggest that from the mid-1970s to 2011 young men were the demographic group of most concern in Greenland, and that their high rates may have stabilised in 1995 [24], data on suicide rates subsequent to 2011 are lacking. Apart from suicide data published by Statistics Greenland reporting 24 suicides among men aged 15–24 in 2012–2013 [5] we lack clear risk estimates enabling temporal trends to be charted. To ensure that current Greenlandic suicide prevention efforts target the groups at highest risk, there is a clear need for updated mortality indicators to be made available so that up-to-date surveillance is directly linked to suicide prevention activity.

Young people were a major focus of Greenland’s 2004 plans for suicide prevention [9], which proposed interventions to improve resilience throughout the education system, and national helplines and radio programmes to overcome barriers such as access to psychological support in remote areas [21,40]. They also proposed strengthening the research base [21], a need reinforced by the findings of this review. The multi-level approaches suggested have been shown to have synergistic benefits [41], but Greenlandic suicide prevention interventions have never been evaluated [42]. Future versions should consider including means restriction, generally understood to be the most effective suicide prevention intervention [43]. The high proportion of violent suicides in Greenland [20], and the historical predominance of hunting/fishing jobs in men who die by suicide [22], suggest a role for gun control interventions such as limiting access to firearms outside working hours. Other issues to address include the impact of labour market changes [10], given qualitative accounts of hopelessness among young men about their opportunities [44]. Evidence from other Arctic Indigenous communities suggests that reform of alcohol policies may not reduce suicides as much as hoped [34]. International collaborations such as the RISING SUN project are likely to be important to Greenland in terms of research infrastructure, and intervention implementation. Implementation of the Canadian Inuit Suicide Prevention Strategy will also be of interest to Greenland, where any successes in Inuit-specific approaches might be emulated [2].

### 4.5. Future Research

Beyond identifying very high suicide rates in young men in Greenland, the studies we identified in our review covered only a very narrow period, providing little detail on how and when age group transitions occurred, or specific suicide risk factors. There is a clear need for studies describing fine-grained temporal changes by age and gender, using appropriate statistical tests, and charting patterns beyond 2011. There is also a need for studies investigating specific risk factors for suicide in young men in Greenland, as those for all age groups and genders are of limited utility in understanding young men. This relies on improved recording of the clinical and socio-demographic characteristics of all suicide cases, with testing for age and gender variation. However, the problem of a lack of power will continue to be an issue in answering specific research questions at the country level. This has been an issue in studies investigating whether there is a differential effect of seasonality in young people in Greenland, where even combining genders was not felt to have overcome the issue of power [20,45]. Seasonality is of interest because of the potential impact of ambient light on sleep patterns, as well as associations with social behavior and working patterns.

Population registers present a valuable opportunity to conduct population-based analysis including routine clinical data, and to describe how risk factors for suicide are distributed demographically. One such study found that young Greenlandic men are more likely than the general Greenlandic population to be diagnosed with a psychiatric disorder [36]. Using suicide data available for the years following 2011, and the potential for linkage with Danish health registers, there is scope to investigate the contribution of psychiatric disorder to suicide risk in different age groups. Primary data collection will be needed to investigate whether seasonal affective disorder, acculturative stress through rapid modernisation, or imitative suicide [46] are contributory factors, as these have not yet been investigated in young men in Greenland. The association between ambient light and suicide rates, the effects of colonial relationships [47] and of climate change, and the influence of the media reporting of suicide on young people in Greenland are also important areas for investigation.

## 5. Conclusions

Our systematic review identified evidence to support a dramatic rise in suicide mortality among Greenlandic men aged 15–24 from the mid-1970s, who then represented the highest risk group from 1976–2011 compared with men and women of all age groups in Denmark and Greenland. However, as no articles investigated risk factors for suicide in young men we lack clear explanations for these disparities. Our findings identify a gap in the evidence describing how and when age group transitions occurred in Greenland, and whether this was related to the rapid social change in Greenland at that time. Our findings also identified the need for studies identifying specific suicide risk factors in young men to inform future suicide prevention strategies.

## Figures and Tables

**Table 1:** Studies included in systematic review (in date order of publication).

Author and Title	Design, Setting and Objectives	Participants	Variables	Results (Those Relevant to Research Question)	Bias and Limitations	Interpretation
Grove and Lynge, 1979 Suicide and attempted suicide in Greenland. A controlled study in Nuuk [19].	Case-control study. Capital of Greenland, Nuuk, in 1972 and 1973. Aimed to identify risk factors for suicide and suicide attempt in cases of fatal and non-fatal suicide attempt compared to general population never suicidal controls.	**Cases**: all Greenlanders in Nuuk who died by suicide or attempted suicide in 1972 and 1973 (n = 1576 males + 1697 females), with attempted suicide defined as that requiring hospital admission. **Controls**: Nuuk residents admitted to hospital for a somatic disease or pregnancy, matched by gender and age group for each case, but with no history of suicide attempt.	Information on suicide cases collected from available records “coming to the attention of any authority in the district of Nuuk.” i.e., death certificates, and police reports. Information on suicide attempt cases was additionally collected from hospital files, police reports, and crime registers to capture unvalidated measures of family composition, childhood, atmosphere in parental home, alcohol consumption, criminal records, exposure to attempted or completed suicide, marital status, inter-racial marriage, psychiatric history, obstetric history, and method of suicide attempt. Age groupings used: 15–19; 20–24; 25–39; ≥40.	Measures presented for young men: absolute numbers of suicide deaths. Findings: 12 suicides recorded in Nuuk during 1972 and 1973: 10 were in men. Age-specific data identified 4 suicides in men aged 15–24 over this period (compared with 2 for women), and 16 suicide attempts in men of the same age. Suicide rates for 4 age-groups were estimated for both genders combined (300 cases of suicide per 100,000 inhabitants in men and women aged 15–19; 173 cases per 100,000 inhabitants in men and women aged 20–24). Suicide rates for men and women aged 25–39 were 226 per 100,000, whilst those for men and women of 40 and over were 54. Risk factors were not separated out by age group or gender. Ratios of suicide attempts to suicide deaths were not presented, but on direct calculation were: 5:3 in men aged 15–19 (compared with 10:1 in similar aged women) 11:1 in men aged 20–24 (compared with 12:1 in similar aged women). 10:5 in men aged 25–39 (compared with 11:0 in similar aged women).	Outcomes were separated out for men and women only in relation to absolute numbers of suicide deaths. Unclear whether method of identifying suicide cases in Nuuk in 1972–1973 was comprehensive. Presented age-specific suicide rates per 100,000 inhabitants (of Nuuk) per year, using census mid-estimates of the population denominator in each age band. Findings from the population of Nuuk may not be generalizable to the whole Greenlandic the population due to urban-rural differences. Estimates of the general population denominator for 1973 were based on mid-point interpolations between 1970 and 1976 census data. These were therefore unreliable as mid-point population estimates would fail to take into account changing migration and fertility patterns.	Little can be concluded about suicide epidemiology in young men in the capital of Greenland as absolute numbers of suicides in men of different age groups were too low to attempt meaningful comparisons. Estimates of age-specific suicide rates were not specific to men, and used an unreliable estimate of the denominator. Direct calculation of the ratios of suicide attempts to suicide deaths indicate that the ratio increases from 5:3 for men aged 15–19 to 11:1 in men aged 20–24 and then falls to 10:5 for men aged 25–39.
Lynge, 1985 Suicide in Greenland [12]	Aggregated data from annual cross-sectional studies of suicide cases. Whole of Greenland during the period 1974–1984. To describe suicide rates in Greenland.	**Cases**: All those aged 15 and above who were born in Greenland and died by suicide in 1974–1984, as identified from death certificates and police reports (n = 318).	Data on age, gender and place of residence of those dying by suicide, based on police reports and death certificates, as well as unvalidated measures of known motives and whether alcohol was involved in the suicide. Age groupings used: 15–19; 20–24; 25–39; ≥40	Measures presented for young men: absolute numbers of suicide deaths; suicide rates per 100,000 inhabitants per year. Findings: 318 total suicides in men and women over this period. 256 were in men, with the highest absolute numbers of suicides (n = 97) in men in the age group 20–24. Age and gender-specific suicide rates per 100,000 inhabitants per year were highest in men age 20–24 (387 per 100,000). The suicide rate in men aged 15–19 (151 per 100,000) was higher than that in men aged 40 and over (73 per 100,000) but lower than that in men aged 20–24 (162 per 100,000). The suicide rates for women were lower than for men in all age groups (44 per 100,000 in women aged 15–19; 94 per 100,000 in women aged 20–24).	Author acknowledges that due to non-systematic collection of death certificates, some cases of suicide may have been missed. Data for an aggregated period obscured year on year changes. Presented crude suicide rates for 100,000 inhabitants per year.	For the period 1974–1984 suicide rates in young men aged 20–24 were the highest of all age and gender groups in Greenland at 387 per 100,000 inhabitants per year, compared with 151 per 100,000 for men aged 15–19, 162 per 100,000 for men aged 25–39, and 73 per 100,000 in men over 40. The gender gap in suicide rates over this period was greatest in the age group 20–24 at 387 *versus* 94 per 100,000. Year on year rates were not presented so no information was provided on temporal trends.
Thorslund, 1990 Inuit Suicides in Greenland [23]	Aggregated data from annual cross-sectional studies of suicide cases. Whole of Greenland from 1977–1986. To describe the epidemiology of suicide among Greenlandic Inuit.	**Cases**: All recorded suicides in Greenland from 1977–1986 among Greenland-born individuals (n = 403), using death certificates and police reports.	Data on age, gender, occupation, marital status, parental status, psychiatric history, substance misuse history, and circumstances of the death, history of suicidal behaviour, derived from public files, death certificates, municipal welfare office records, and police reports. Age groupings used: histogram presented data for a continuous measure of age from 10 to 60	Measures presented for young men: average suicide rates per 100,000 population per year. Findings: 403 suicides analysed over a 10 year period, of which 326 were of men (of any age). Visual plots of average suicide rates per 100,000 population per year showed that suicide risk increased sharply from the ages of approximately 15–17, peaking at approximately age 20, falling from approximately age 23, and with further peaks in the mid-30s and the 50s. The great disparity between male and female suicide rates from the age of about 17 diminished by approximately age 27. Data for risk factors, including occupation, was not separated out by gender or age group.	Little information was presented regarding the reliability of routine data. Data for an aggregated period obscured year on year changes. Presented crude suicide rates for 100,000 inhabitants, as an average per year using data over a 10 year period 1977–1986. Data for risk factors was not presented by gender or age group.	Over the 10 year period 1977–1986, men aged approximately 20 to 23 had the highest suicide rates in the Greenlandic population, greatly exceeding those for women of the same age.
Thorslund, 1991Suicide Among Inuit Youth in Greenland 1977–1986 [22]	Aggregated data from annual cross-sectional studies of suicide cases. Whole of Greenland from 1977–1986. To describe risk factors for suicide among youths aged 15–30 in Greenland.	**Cases**: All suicides in Greenland from 1977–1986 of youths aged 15–30 (n = 287), using death certificates and police reports. **Controls**: n = 320 randomly selected young people living in Greenland, sampled via postal questionnaire in 1988.	Data collected on place of residence (town/village), occupation, presence of alcohol in blood, and personal circumstances prior to death, based on public files (police reports, death certificates and social service departments), Public Databases (Public Health Department and Central Bureau of Statistics) and from questionnaires (for controls). Age groupings used: 15–30.	Measures presented for young men: crude average number of suicides per year per 100,000 inhabitants (for both genders); proportions of suicide deaths by gender and occupational group (aged 15–30); proportions of suicide deaths by gender and recent psychosocial stressor (all ages). Findings: In a sub-sample of youth dying by suicide aged 15–30 in 1982–1986 for which occupational data were presented (n = 184), the majority were men (n = 144; 78%). Of this sub-sample, 26% of men were hunters/fishermen *versus* 0% of women (and 15% of male controls), 21% were unskilled workers vs 13% of women (and 37% of male controls), 24% were unemployed vs 38% of women (and 3% of male controls), and 5% were white collar workers vs 10% of women (and 13% of male controls). No statistical tests were presented for these comparisons. For men and women dying by suicide aged 15–30 from 1977–1986, in over 30% of cases there were reports of personal problems with a spouse prior to the suicide, with the text indicating that this was more common in women (but not whether this was statistically significant). The text also indicated that in 20% of male suicides there was a history of rejection from parents or friends “or other shameful situations” prior to the suicide.	Data for an aggregated period obscured year on year changes. Little information presented regarding the reliability of routine data. Very little information provided on the characteristics of the control group used in the comparison of proportions of men and women dying by suicide by occupational group (derived from a survey of Greenlandic residents in 1988). No statistical tests presented for comparisons between men and women aged 15–30, or between cases and controls. Data on risk factors were not presented by gender. Presented crude average number of suicides per year per 100,000 inhabitants (for both genders).	From 1982–1986, 78% of suicides among youth aged 15–30 were in men. These men primarily worked in traditional hunting/fishing jobs, unskilled jobs, or were unemployed. On the basis of cases of suicide in those aged 15–30 from 1977–1986, rejection by parents of friends or “other shameful situations” were implicated in 20% of male suicide cases aged 15–30.
Leineweber & Arensman 2003 Culture Change and Mental Health: The Epidemiology of Suicide in Greenland [24]	Aggregated data from annual cross-sectional studies of suicide cases. Whole of Greenland from 1972–1995, with age- and gender-specific data only presented for the years 1990–1995. To describe suicide rates in Greenland.	**Cases**: All suicides in Greenland from 1972–1995 of Greenland-born individuals, using the register of causes of death based on death certificates certified by a physician using ICD code diagnoses.	Data collected on gender, age and place of residence were drawn from a computerized register on causes of death for persons born in Greenland. Population suicide rates were age-standardised by direct standardisation. Age groupings used: histograms presented plots for ages 10–15; 15–19; 20–24; 25–29; 30–39; 40–49; 50–59; 60+ (or 15–19; 20–24; 25–29; ≥30)	Measures presented for young men: Visual plots of suicide rates per 100,000 population by sex and age-group. Findings: Visual plotting of suicide rates per 100,000 population by age-group and sex in Greenland in the period 1990–1995 showed that rates in men aged 15–24 were the highest of all age-groups for either gender; approximately 460 per 100,000 population for those aged 15–19, declining with age, but remaining above 400 per 100,000 for those aged 20–24, and approximately 300 per 100,000 for men aged 25–29. Rates for men were higher than for women in all age groups. Risk factors for suicide were not separated out by age-group or gender.	Data for an aggregated period obscured year on year changes. Risk factors for suicide were not separated out by age-group or gender.	From 1990–1995 suicide rates in men aged 15–19 and 20–24 were the two highest ranking age groups for all age-groups in either gender. Rates for men aged 15–24 were approximately 460 per 100,000, and declined with age, remaining higher for men than women in all age groups.
Bjorksten, Bjerregaard et al., (2005) Suicides in the midnight sun—A study of seasonality in suicides in West Greenland [20]	Suicide data from annual cross-sectional studies of suicide cases only for West Greenland, 1968–1995, with rates for 7 specific years reported. To investigate whether there is evidence for seasonality of suicide rates in West Greenland.	**Cases**: Suicides of people of any age living in towns and settlements in West Greenland, as recorded in the register of causes of death in Greenland, and population registers from the National Institute of Public Health in Copenhagen were analysed (n = 833).	Data collected on age, gender, suicide date, country of birth (Greenland/Denmark), residence (town/settlement), latitude, and whether alcohol contributed to their death, based computerised registers on causes of death in Greenland and population registers from the National Institute of Public Health in Copenhagen. Age groupings used: 0–14; 15–24; 25–34; 35–59; ≥60.	Measures presented for young men: suicide rates per 100,000 person-years by age-group; seasonality of suicides by gender (men of all ages) and age group (all men and women ≤ 24 vs. >24). Findings: Of 684 total suicides in men over the period 1968 to 1995, the median age of cases was 25 and the age range was 11 to 84 years. When considering the specific years 1970, 1976, 1982, 1987, 1990, 1993, and 1995 (for which detailed population data were available, allowing calculation of suicide rates per 100,000 person-years), results showed that men aged 15–24 had the highest suicide rates between 1976 and 1995, peaking at 577 per 100,000 person-years in 1990. For the period 1970 to 1995, the longitudinal picture for male suicide rates was one of great change. In 1970, all 122 suicide cases had been men aged 35–59 years of age, with the overall suicide rate for men of all ages at 22 per 100,000 inhabitants per year. From 1976 to 1995, the overall suicide rate in men climbed from 80 per 100,000 in 1976 to a maximum value of 214 per 100,000 in 1990. For young men aged 15–24, suicide rates climbed from 0 in 1970 to 249 per 100,000 in 1976 to a maximum of 577 per 100,000 in 1990. This age group had the highest suicide rates of all male age groups during 1976–1995, plateauing at 238 per 100,000 in 1995. Men of 25–34 years of age also had high suicide rates from 1976 on, peaking at 297 per 100,000 in 1990. In 1995 suicide rates for men aged 15–24 remained the highest at 238 per 100,000, but disparities with those for men aged 25–34 were less at 219 per 100,000 person-years. Analysis of seasonality of suicides was examined for men (all ages), finding a statistically significant midsummer peak, and for men and women combined aged ≤ 24, finding no significant seasonality. Risk factors for suicide were not broken down by age-group or gender.	Estimates of suicide rates by age group for specific years over the period 1968–1995 may not be generalisable to East Greenland or Nuuk, where suicide rates tended to be higher in the 1970s and 1980s. Suicide rates presented as actual numbers of suicides per year for the period 1968–1995, and as crude rates per 100,000 person-years for the specific years 1970, 1976, 1982, 1987, 1990, 1993, and 1995. The finding of no significant seasonality by age may have been due to low numbers	No observations can be made about seasonality of suicide in young men specifically, but there was a significant midsummer peak for men of all ages. For young people, no seasonality was observed in men and women (combined) aged 24 and under for the period 1968–1995. In the whole population, significant seasonality was observed, with a peak in late June and lowest rates in the darkest months (December to February). From 1976–1995 suicide rates were highest in men aged 15–24, peaking at 577 per 100,000 in 1990. In 1995 they remained the highest for all male age groups at 238 per 100,000 but with less of a disparity with rates for men aged 25–34.
Bjerregaard and Lynge (2006) Suicide—A Challenge in Modern Greenland [21]	Aggregated data from annual cross-sectional studies of suicide cases. Whole of Greenland from 1968–1999. To describe suicide rates in young men in Greenland in 1968–1999, compared with those in other demographic groups. Study also presented linked cross-sectional population-based survey data from 2 population surveys in Greenland (in 1993–1994 and 1999–2001) describing past year prevalence of suicidal thoughts (not meeting search criteria for the current study but brief details given here).	**Cases**: All suicides in Greenland from 1968–1999 of Greenland-born individuals (n = 1203), using the register of causes of death based on death certificates certified by a physician using diagnoses based on ICD-8 (1968-1993) or ICD-10 (1994–1999).	Data collected on age, gender, and place of residence. Age groupings used: histogram presented data for a continuous measure of age from 0 to approximately 85, with points specified for 0–4; 10–14; 20–24; 30–24; 40–44; 50–54; 60–64; 70	Measures presented for young men: visual plots of age-specific suicide rates per 100,000 person-years; visual plots of youth suicide rates (aged 15–29) for men per 100,000 births in each cohort. Findings: Overall for the period 1968–1999 and all age groups, suicide rates were 4.3 times higher in men than women. Visual plots of age-specific suicide rates for the aggregated period 1990–1999 showed suicide rates to be considerably higher in men aged 15–24 in Greenland (approximately 470 per 100,000 person-years) than for men of a similar age in Denmark and for women of a similar age in Greenland and in Denmark. This disparity continued up until approximately the age 45, when rates in all four groups converged. Youth suicide rates (aged 15–29) for men in the 1950 birth cohort were plotted as approximately 10 per 100,000 births in that cohort, rising to approximately 60 per 100,000 births in the 1978 birth cohort. Although gender-specific data were not presented, visual plots showed that youth suicide rates (aged 15–29) in East Greenland continued to rise from 1975–1979 (approximately 200 per 100,000 person years) to 1995–1999 (approximately 800 per 100,000 person years), in comparison to West Greenland and the capital Nuuk where they plateaued or fell slightly (to between 100 and 200 per 100,000 person years in 1995–1999). Other risk factors for suicide were not broken down by age-group or gender. Additional analysis of data from population surveys called out in 1993–1994 and 1999–2001 showed that men aged 18–24 were significantly less likely that same-aged women to report lifetime suicidal thoughts (19% *versus* 33%; p = 0.03). At age groups above 25 this gender difference was not statistically significant.	Figures in text did not match those in graphical presentations. Data for an aggregated period obscured year on year changes. Suicide rates presented as crude rates per 100,000 person-years for blocks of 5 years, or as crude rates per 100,000 births in a birth cohort.	Aggregated data for 1990–1999 show that suicide rates in young men aged 15–24 were much higher than those for men in other age groups and for men and women of the same age in Denmark, peaking at 450–500 per 100,000 person-years. This pattern differed from that in Denmark, where suicide rates rose across the age groups for men and women, and where a much lower ratio of male:female suicides was reported (1.8 in Denmark *versus* 4.3 in Greenland). There was evidence that male youth suicide rates (in the age group 15–29) were higher in men born in later cohorts (downstream of sociocultural change), as evidenced in data from cohorts born from 1950 to 1978. Regional variations in youth suicide were not broken down by gender, but suggested that young people in remote areas of East Greenland had higher suicide rates, which continued to rise, whilst youth suicide rates had peaked in the capital, Nuuk, in the early 1980s, and plateaued in West Greenland throughout the 1980s and 1990s. Population survey data from 1993–1994 and 1999–2001 suggested that although suicide rates in young men aged 15–24 were considerably higher than those for women of that age in Greenland, women were significantly more likely to report lifetime suicidal thoughts.
Bjerregaard & Larson (2015) Time trend by region of suicides and suicidal thoughts among Greenland Inuit [18]	Aggregated data from annual cross-sectional studies of suicide cases, averaging out rates over periods of between 5 and 30 years Study also presented linked cross-sectional population-based survey data from 2 population surveys in Greenland (in 1993–1994 and 2005–2010) describing past year prevalence of suicidal thoughts (not meeting search criteria for the current study but brief details given here). Whole of Greenland from 1970–2011.To describe time trends in suicide rates (and past year prevalence of suicidal thoughts in 1993–1994 and 2005–2010) in Greenland from 1970 to 2011	**Cases**: All suicides in Greenlandic residents from 1901–2011 (n = 1678), based on routine registry data from the Greenland registry of causes of death. General population sample of Greenlandic residents sampled 1993–1994 and 2005–2010 using the same instrument, and overlapping geographical sampling frames, to collect data on self-reported past year prevalence of suicidal thoughts, with linkage of individuals in cross-sectional surveys to subsequent suicides.	Data collected on age, gender, and region of residence for all suicide cases from 1901–2011. Survey data collected on age, gender, and past year prevalence of suicidal thoughts in two cross-sectional samples. Age groupings used: 10–14; 15–19; 20–24; 25–29; 30–34; 35–44; 45–54; ≥55	Measures presented for young men: suicide rates per 100,000 person-years by age-group (tabulated and visual plots) Findings: Suicide rates for men aged 20–24 were the highest of all male age groups for the period 2000–2011 (at 426 per 100,000 person-years over this whole 12 year period), compared with 297 per 100,000 person years for men aged 15–19 and 251 for men aged 25–29. Suicide rates for men aged 55 and above were the lowest of all male age groups (at 74 per 100,000 person years). Visual plots of suicide rates for men and women by age group for the period 1970 to 2011 also show that rates were highest for men aged 20–24 (at approximately 400 per 100,000 person years). NB: We clarified with the first author that the suicide data presented in his table II are from the registry of causes of death and cover 2000–2011 only, to be comparable to the survey data on suicidal thoughts. He confirmed that the suicide data presented in Figure 1 are also derived from the registry of causes of death but cover the whole time range of the registry, which at that time was 1970–2011. There is hence some overlap between the data from these two sources, and the age pattern was similar in the shorter period and over the whole period. Age patterning of high risk groups for past-year suicidal thoughts mirrored that for suicide rates in men, with the highest rate in men aged 20–24 at 136 per 1000 participants. The proportion of women with past-year suicidal thoughts was greatest for women aged 15–19 (188 per 1000 participants) and fell with increasing age. Age- and gender-specific suicide rates were not presented by region of residence, but the text indicated that male and female patterns were similar to the overall pattern. This pattern, since the 1980s, has been for suicide rates for men, women, and overall to have been significantly higher in East and North Greenland than in West Greenland or the capital, Nuuk. Association between past year suicidal ideation and subsequent suicide was not presented by gender or age group.	Data for an aggregated period obscured year on year changes. Suicide rates presented as crude rates per 100,000 person-years for blocks of between 5 and 30 years. Source and quality of data from 1970–2011 not qualified. Due to small absolute numbers of completed suicides, there was insufficient statistical power to stratify analyses on several levels simultaneously e.g., sex and region. Unclear whether survey instrument validated. [Respondents in the two surveys, and within the same sampling period, could have been sampled twice. No discussion of response rate, recall bias, or social desirability bias.]	Suicide rates for men aged 20–24 were the highest of all age groups for both genders over the 41 year period 1970 to 2011 (at approximately 400 per 100,000 person years), and when considered over the shorter 12 year period of 2000–2011 (at 426 per 100,000 person-years). Age groups at highest risk of suicide match those at highest risk of suicidal thoughts for men.

## References

[1] Pitman A., Krysinska K., Osborn D., King M. (2012). Suicide in young men. Lancet.

[2] Collins P.Y., Delgado R.A., Pringle B.A., Roca C., Phillips A. (2017). Suicide prevention in Arctic indigenous communities. Lancet Psychiatry.

[3] Young T.K., Revich B., Soininen L. (2015). Suicide in circumpolar regions: An introduction and overview. Int. J. Circumpolar Health.

[4] Moltke I., Fumagalli M., Korneliussen T.S., Crawford J.E., Bjerregaard P., Jørgensen M.E., Grarup N., Gulløv H.C., Linneberg A., Pedersen O. (2015). Uncovering the genetic history of the present-day Greenlandic population. Am. J. Hum. Genet..

[5] StatbankGreenland National Board of Health Mortality Statistics 1990–2013. http://bank.stat.gl/.

[6] WHO Country Reports and Charts. http://www.who.int/topics/suicide/en/.

[7] WHO Global Health Observatory (GHO) Data: Suicide Rates per (100,000 Population). http://www.who.int/gho/mental_health/suicide_rates_crude/en/.

[8] White A., Holmes M. (2006). Patterns of mortality across 44 countries among men and women aged 15–44 years. J. Mens Health Gender.

[9] PAARISA (2004). Proposal for a National Strategy for the Prevention of Suicides in Greenland.

[10] Bjerregaard P., Larsen C.V. (2018). Three lifestyle-related issues of major significance for public health among the Inuit in contemporary Greenland: A review of adverse childhood conditions, obesity, and smoking in a period of social transition. Public Health Rev..

[11] Bertelsen A. (1935). Gronlandsk medicinsk statistik og nosografi (medical statistics and nosography of Greenland). Meddl. Gronland.

[12] Lynge I. (1985). Suicide in Greenland. Arct. Med. Res..

[13] Kapur N., Cooper J., O’Connor R.C., Hawton K. (2013). Non-suicidal self-injury v. Attempted suicide: New diagnosis or false dichotomy?. Br. J. Psychiatry.

[14] Cooper J., Kapur N., Webb R., Lawlor M., Guthrie E., Mackway-Jones K., Appleby L. (2005). Suicide after deliberate self-harm: A 4-year cohort study. Am. J. Psychiatry.

[15] Liberati A., Altman D.G., Tetzlaff J., Mulrow C., Gøtzsche P.C., Ioannidis J.P.A., Clarke M., Devereaux P.J., Kleijnen J., Moher D. (2009). The PRISMA statement for reporting systematic reviews and meta-analyses of studies that evaluate healthcare interventions: Explanation and elaboration. BMJ.

[16] Elm E.v., Altman D.G., Egger M., Pocock S.J.P., Gøtzsche P.C., Vandenbroucke J.P. (2007). Strengthening the reporting of observational studies in epidemiology (strobe) statement: Guidelines for reporting observational studies. BMJ.

[17] Moher D., Liberati A., Tetzlaff J., Altman D.G. (2009). Preferred reporting items for systematic reviews and meta-analyses: The PRISMA statement. PLoS Med..

[18] Bjerregaard P., Larsen C. (2015). Time trend by region of suicides and suicidal thoughts among Greenland Inuit. Int. J. Circumpolar Health.

[19] Grove O., Lynge J. (1979). Suicide and attempted suicide in Greenland. A controlled study in Nuuk (godthaab). Acta Psychiatr. Scand..

[20] Bjorksten K.S., Bjerregaard P., Kripke D.F. (2005). Suicides in the midnight sun--a study of seasonality in suicides in West Greenland. Psychiatry Res..

[21] Bjerregaard P., Lynge I. (2006). Suicide—A challenge in modern Greenland. Arch. Suicide Res..

[22] Thorslund J. (1991). Suicide among Inuit youth in Greenland 1977–1986. Arct. Med. Res..

[23] Thorslund J. (1990). Inuit suicides in Greenland. Arct. Med. Res..

[24] Leineweber M., Arensman E. (2003). Culture change and mental health: The epidemiology of suicide in Greenland. Arch. Suicide Res..

[25] Blanchard R.D., Bunker J.B., Wachs M. (1977). Distinguishing aging, period and cohort effects in longitudinal studies of elderly populations. Socio-Econ. Plan. Sci..

[26] World Health Organization (WHO) (2002). Multisite Intervention Study on Suicidal Behaviours-Supre-Miss: Protocol of Supre-Miss.

[27] Box J.E. (2002). Survey of Greenland instrumental temperature records: 1873–2001. Int. J. Climatol..

[28] Government of Greenland Politics in Greenland. http://naalakkersuisut.gl/en/About-government-of-greenland/About-Greenland/Politics-in-Greenland.

[29] Bjerregaard P. (2001). Rapid sociocultural change and health in the Arctic. Int. J. Circumpolar Health.

[30] Leighton A.H., Hughes C.C. (1955). Notes on Eskimo patterns of suicide. Southwest. J. Anthropol..

[31] Silviken A., Haldorsen T., Kvernmo S. (2006). Suicide among indigenous Sami in Arctic Norway, 1970–1998. Eur. J. Epidemiol..

[32] EchoHawk M. (2006). Suicide prevention efforts in one area of Indian health service, USA. Arch. Suicide Res..

[33] Boothroyd L.J., Kirmayer L.J., Spreng S., Malus M., Hodgins S. (2001). Completed suicides among the Inuit of northern Quebec, 1982–1996: A case-control study. CMAJ Can. Med. Assoc. J..

[34] Berman M. (2014). Suicide among young Alaska native men: Community risk factors and alcohol control. Am. J. Public Health.

[35] Qin P. (2005). Suicide risk in relation to level of urbanicity—A population-based linkage study. Int. J. Epidemiol..

[36] Lynge I., Mortensen P.B., Munk-Jorgensen P. (1999). Mental disorders in the Greenlandic population. A register study. Int. J. Circumpolar Health.

[37] Thorslund J., Misfeldt J. (1989). On suicide statistics. Arct. Med. Res..

[38] Bjerregaard P., Curtis T. (2002). Cultural change and mental health in Greenland: The association of childhood conditions, language, and urbanization with mental health and suicidal thoughts among the Inuit of Greenland. Soc. Sci. Med..

[39] Broderstad A.R., Eliassen B.M., Melhus M. (2011). Prevalence of self-reported suicidal thoughts in SLiCA. The survey of living condition in the Arctic (SLiCA). Glob. Health Action.

[40] Le Fevre A.C. (2004). The challenge of reducing youth suicide in Greenland—Interventions, strategies and roads to be explored. Int. J. Circumpolar Health.

[41] Van der Feltz-Cornelis C.M., Sarchiapone M., Postuvan V., Volker D., Roskar S., Grum A.T., Carli V., McDaid D., O’connor R., Maxwell M. (2011). Best practice elements of multilevel suicide prevention strategies. Crisis.

[42] Redvers J., Bjerregaard P., Eriksen H., Fanian S., Healey G., Hiratsuka V., Jong M., Larsen C.V.L., Linton J., Pollock N. (2015). A scoping review of indigenous suicide prevention in circumpolar regions. Int. J. Circumpolar Health.

[43] Zalsman G., Hawton K., Wasserman D., van Heeringen K., Arensman E., Sarchiapone M., Carli V., Höschl C., Barzilay R., Balazs J. (2016). Suicide prevention strategies revisited: 10-year systematic review. Lancet Psychiatry.

[44] Soule S. (2008). An Evaluation of the Implementation of Greenland’s National Strategy for Suicide Prevention with Recommendations for the Future.

[45] Björkstén K.S., Bjerregaard P. (2015). Season of birth is different in Inuit suicide victims born into traditional than into modern lifestyle: A register study from Greenland. BMC Psychiatry.

[46] Leineweber M., Bjerregaard P., Baerveldt C., Voestermans P. (2001). Suicide in a society in transition. Int. J. Circumpolar Health.

[47] Bolliger L., Gulis G. (2018). The tragedy of becoming tired of living: Youth and young adults’ suicide in Greenland and Denmark. Int. J. Soc. Psychiatry.

